# Supplementary material for: Complex‐centric proteome profiling by SEC‐SWATH‐MS
Source: Mol Syst Biol. 2019 Jan 14;15(1):e8438. doi: 10.15252/msb.20188438 (PMC6346213; doi:10.15252/msb.20188438)
Supplement: Supplementary file 8 — Dataset EV7 [file MSB-15-e8438-s008.zip › feature_plots_string/P03897.pdf]

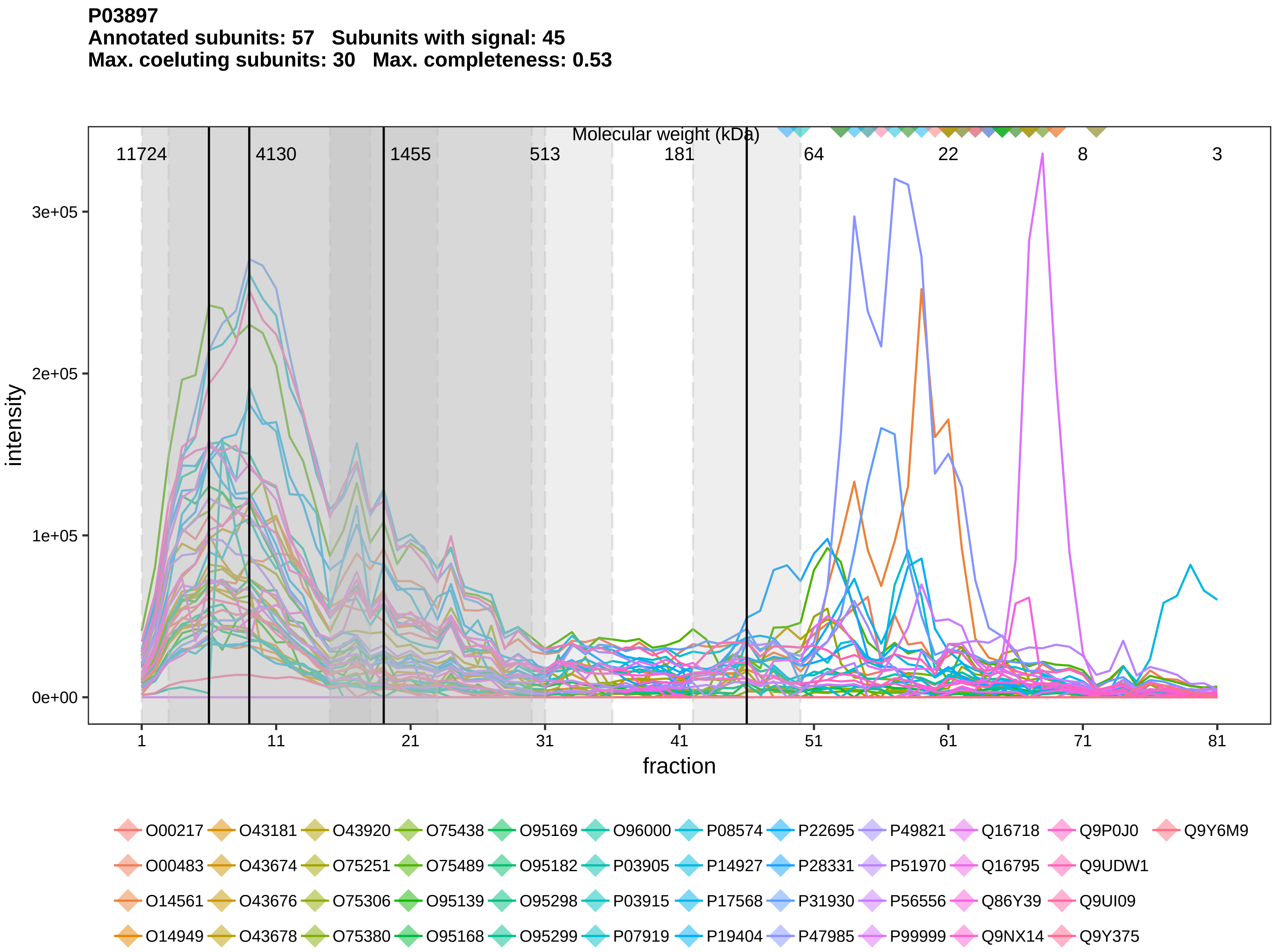

P03897
Annotated subunits: 57 Subunits with signal: 45
Max. coeluting subunits: 30 Max. completeness: 0.53
Molecular weight (kDa)
11724
4130
1455
513
181
64
22
8
3
3e+05
2e+05
1e+05
0e+00
intensity
1
11
21
31
41
51
61
71
81
fraction
O00217
O43181
O43920
O75438
O95169
O96000
P08574
P22695
P49821
Q16718
Q9P0J0
Q9Y6M9
O00483
O43674
O75251
O75489
O95182
P03905
P14927
P28331
P51970
Q16795
Q9UDW1
O14561
O43676
O75306
O95139
O95298
P03915
P17568
P31930
P56556
Q86Y39
Q9UI09
O14949
O43678
O75380
O95168
O95299
P07919
P19404
P47985
P99999
Q9NX14
Q9Y375
